# Segregation and Morphological Evolution of Si Phase during Electromagnetic Directional Solidification of Hypereutectic Al-Si Alloys

**DOI:** 10.3390/ma12010010

**Published:** 2018-12-20

**Authors:** Weiyan Jiang, Wenzhou Yu, Jie Li, Zhixiong You, Chunmei Li, Xuewei Lv

**Affiliations:** 1 College of Materials Science and Engineering, Chongqing University, Chongqing 400045, China; jwy1020@163.com (W.J.); lijie04job@163.com (J.L.); youzx@cqu.edu.cn (Z.Y.); may840@cqu.edu.cn (C.L.); lvxuewei@163.com (X.L.); 2 Chongqing Key Laboratory of Vanadium-Titanium Metallurgy and Advanced Materials, Chongqing University, Chongqing 400044, China

**Keywords:** electromagnetic directional solidification, Al-Si alloy, primary Si, macrosegregation mechanism, morphological evolution

## Abstract

Understanding the Si segregation behavior in hypereutectic Al-Si alloys is important for controlling the micro- and macrostructures of ingots. The macrosegregation mechanism and morphological evolution of the primary Si phase were investigated during electromagnetic directional solidification (EMDS). Both numerical simulations and experimental results strongly suggested that the severe macrosegregation of the primary Si phase was caused by fluid flow and temperature distribution. Microscopic analysis showed that the morphological evolution of the Si crystal occurred as follows: planar → cellular → columnar → dendritic stages during EMDS. Based on constitutional supercooling theory, a predominance area diagram of Si morphology was established, indicating that the morphology could be precisely controlled by adjusting the values of temperature gradient (*G*), crystal growth rate (*R*), and solute concentration (*C*_0_). The results provide novel insight into controlling the morphologies of primary Si phases in hypereutectic Al-Si alloys and, simultaneously, strengthen our understanding of the macrosegregation mechanism in metallic alloys.

## 1. Introduction

Macrosegregation often occurs during the solidification of metallic materials and can significantly deteriorate the mechanical properties of the final ingots [1]. Controlling or minimizing macrosegregation is commercially important as it can reduce material losses during casting. Despite over a century of research on macrosegregation and a good understanding of the underlying mechanisms, the relationship between the mechanisms and structure formed during solidification remains unclear [2].

It is recognized that fluid flow, arising from either natural or forced convection, can profoundly affect macrosegregation [3]. Natural convection (i.e., thermosolutal convection) is driven by differences in the densities and temperatures of liquid melts. Forced convection, on another hand, often arises from mechanical, electromagnetic, or other types of stirring. An intense convection can always result in solute redistribution and the relative movement of solid and liquid phases, leading to a nonuniform structure of the alloy. In addition to fluid flow, solidification conditions including thermal gradients (*G*), solidification rates (*R*), and solute contents (*C*) can also impact macrosegregation and crystal growth [4]. Microstructures are actually the results of the complex coupling of fluid flow, heat transfer, solute distribution, and phase transitions. Hence, understanding the roles of each of these solidification parameters would be beneficial for controlling the final ingot microstructures.

Hypereutectic Al-Si alloys are typical materials in which the primary Si phase often shows severe macrosegregation in large ingots. Under the conventional solidification process, the primary Si phases usually exhibit a variety of morphologies including polygonal [5], star-like [6], and coarse platelet structures [7]. Inhomogeneous structure and coarse primary Si phase cause deterioration in alloy machinability, thereby significantly limiting the widespread application of hypereutectic Al-Si alloys. The macrosegregation behavior of Al-Si alloys has been studied in recent years. For example, Kang et al. [8] showed that the macrosegregation was significantly influenced by the size and temperature of the molten pool in selective laser melting process. Ghods et al. [9] proved that the extent of macrosegregation decreased with increasing the crystal growth speed during the directional solidification. To date, methods such as electromagnetic stirring [10], ultrasonic vibration [11], rapid cooling [12], and modifier addition [13] have been developed to further homogenize solute distributions and refine grains. Electromagnetic stirring is a desirable candidate because it is a noncontact method that does not contaminate the alloy. Owing to this, the effects of electromagnetic stirring on grain refinement have been widely concerned. Robels-Hernández et al. [14] demonstrated that electromagnetic stirring and vibration (ESV) could modify the primary Si particles by transforming them into “eutectic-like” particles at temperatures close to the liquidus (i.e., 620 °C for the 390 Al-Si alloy). Yoshiki et al. [15] showed that applying ~1 KHz electromagnetic vibration could effectively refine silicon particles up to 5 μm in diameter. These indicate that the electromagnetic stirring can effectively refine the Si particles. However, opposing viewpoints have also been put forward by Jie et al. [16] who demonstrated that although an intense rotating magnetic field (RMF) was employed, the Si agglomeration phenomenon was observed as the solidification rate was slow. This implies that the formation and distribution of Si phase in hypereutectic Al-Si alloys should be determined by many factors, other than single factor.

In addition to the important issue of refining Si particles, many studies [17,18,19] have been conducted concerning the enrichment of the primary Si phase from hypereutectic Al-Si alloys. The enriched Si can subsequently be used for preparing high-purity Si. If a planar Si morphology (bulk Si) could be realized by controlling solidification conditions, the manufacturing cost of high-purity Si could be significantly reduced. However, the dependence of Si crystal growth on solidification parameters such as fluid flow intensity, cooling rate, temperature gradient, and solute concentration remains unclear. Additionally, Si morphological evolution under various solidification conditions requires further research.

In this study, an electromagnetic directional solidification (EMDS) procedure was developed to reveal the formation mechanism of the primary Si phase in hypereutectic Al-Si alloys. The influence of various solidification parameters on the evolution of Si-phase morphology was also investigated herein.

## 2. Materials and Methods

Hypereutectic Al-Si alloys were prepared by mixing high-purity Al pellets (99.997 wt.% Al) and metallurgical Si powder (99.8 wt.% Si) in a high-purity dense graphite crucible (outer diameter = 26 mm; inner diameter = 20 mm; depth = 90 mm). The mixtures were then melted to form alloys in an electrical resistance-heating furnace. By adjusting the mass percentages of Si powder and Al pellets, Al-35 wt.% Si, Al-45 wt.% Si, Al-55 wt.% Si, and Al-65 wt.%, Si hypereutectic alloys were prepared and the total weight of each sample was 30 g.

**Figure 1:**
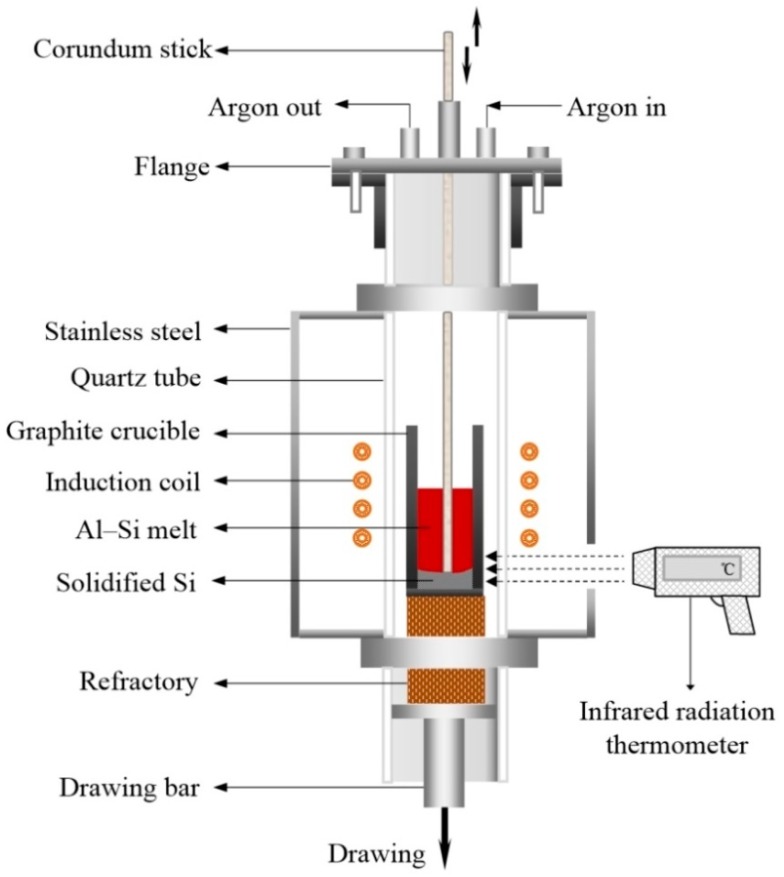
Schematic of the electromagnetic directional solidification setup.

Figure 1 shows a schematic of the EMDS setup. A 60 kW high-frequency induction furnace (Huayang GZP, Zhuzhou, China) equipped with an induction coil (outer diameter = 70 mm; height = 65 mm) was used to heat the samples. The hypereutectic Al-Si alloys were placed in the furnace with the bottoms of the alloys level with the lower position of the induction coils. To completely melt the samples, the current intensity of the induction coil was maintained at 10 A and the samples were held at 1200 ± 5 °C for 30 min. The holding temperature was measured using an infrared radiation thermometer. The melted samples were slowly drawn (at 5 μm/s) out of the heating zone using the drawing system to develop a temperature gradient along the axial direction of the samples, as shown in Figure 1. The temperature gradient of the sample was measured using the infrared radiation thermometer at various positions.

The primary Si phase gradually congregated at the bottom of the sample, forming a Si-rich layer, and the solid-liquid interface axially migrated from the lower to the upper part of the sample until complete solidification. To accurately estimate the migration velocity of the solid-liquid interface, a corundum stick was inserted into the melt to detect the solidified Si layer in the lower part of the sample. Every time the sample was drawn 0.5 cm, the height of the solidified Si layer was measured. When the primary Si phase had completely precipitated in the lower part of the sample, the height of the solidified Si layer remained constant.

The solidified samples were longitudinally cut, ground using SiC paper, and polished to examine the micro- and macrostructures of the primary phase Si-enriched crystals using an Olympus PME3 light optical microscope (Carl Zeiss, Shanghai, China) equipped with a KAPPA image analyzer (Valmet, Espoo, Finland) and Sony digital camera (SONY, Tokyo, Japan).

## 3. Results

**Figure 2:**
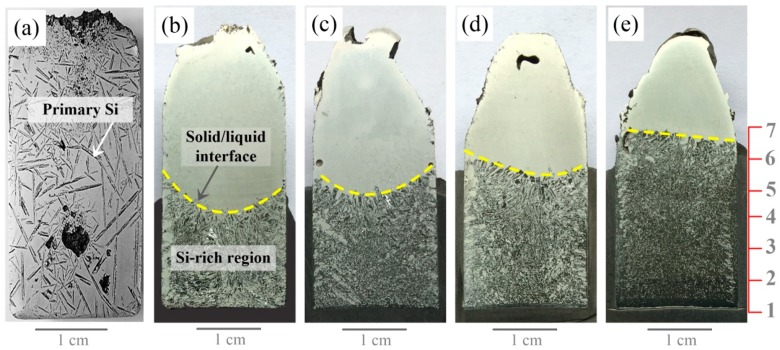
Cross-sections of the solidified samples: (**a**) Al-35 wt.% Si without EMDS, (**b**) Al-35 wt.% Si with EMDS, (**c**) Al-45 wt.% Si with EMDS, (**d**) Al-55 wt.% Si with EMDS, and (**e**) Al-65 wt.% Si with EMDS.

Figure 2 shows the cross-sections of the solidified samples with and without EMDS, respectively. It can be observed from Figure 2a that the needle-like primary Si distributes randomly in the Al-Si alloy when the EMDS was not employed. However, the primary Si particles clearly congregated in the lower parts of the samples as the EMDS was used, as shown in Figure 2b–e. The separation interface was observed at the middle part of each sample. Above the separation interface, almost no primary phase Si crystals were observed, indicating that the primary Si phase severely macrosegregated during solidification. In addition, the height of the Si-rich layer and shape of the separation interface (i.e., solid-liquid interface) changed with varying initial composition of the hypereutectic Al-Si alloy. The Al-35 wt.% Si sample exhibited the shortest Si-rich layer, and the separation interface appeared semicircular (Figure 2b). With increasing Si content, the height of the Si-rich layer increased and the shape of the separation interface gradually flattened (Figure 2e).

**Figure 3:**
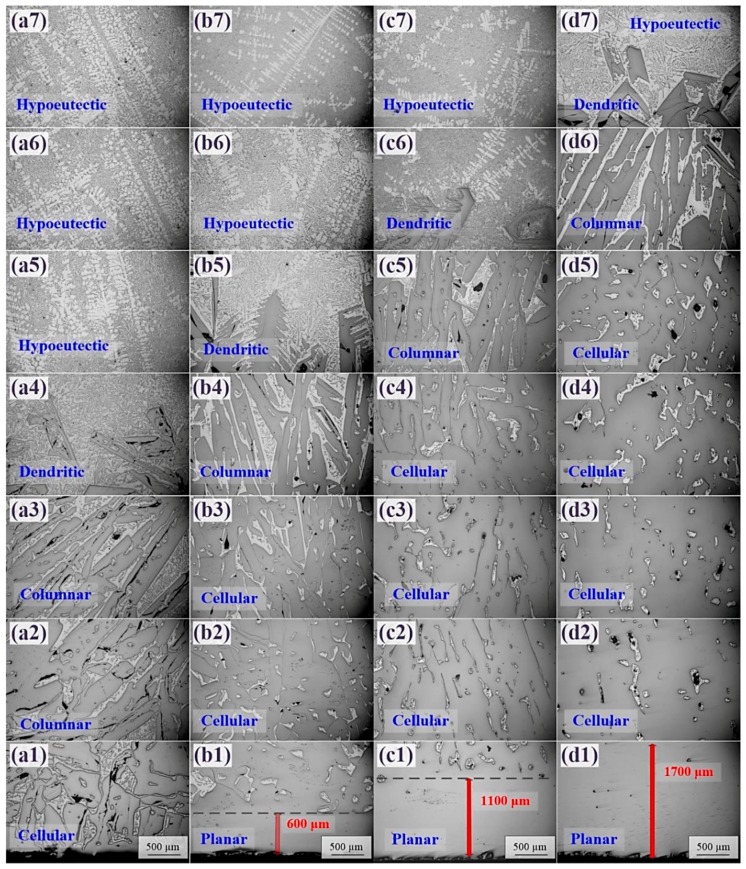
Microstructures of the solidified alloys with EMDS: (**a**) Al-35 wt.% Si, (**b**) Al-45 wt.% Si, (**c**) Al-55 wt.% Si, and (**d**) Al-65 wt.% Si.

To further analyze the segregation behavior of the primary Si phases, the microstructures of alloys with various Si contents were examined, and the results are shown in Figure 3. It should be noted that the microstructures in Figure 3 were selected from seven consecutive positions spaced vertically 0.5 cm apart in the directionally solidified samples, as indicated by the numbers in Figure 2.

Figure 3 shows that the microstructure of the primary Si phases (dark) changed gradually from the bottom to the top of the samples. A planar Si morphology was observed at the bottom, as shown in Figure 3b1,c1,d1. The heights of the planar Si in the Al-45 wt.% Si, Al-55 wt.% Si, and Al-65 wt.% Si samples were ~600, ~1100, and >1700 μm, respectively, indicating that bulk Si was obtained during EMDS of the hypereutectic Al-Si alloy. Furthermore, the morphological evolution of the Si growth interface in the Si-rich region, moving from bottom to top, transitioned through planar → cellular → columnar → and dendritic stages during EMDS. Above the Si-rich regions, the microstructures exhibited typical features of hypoeutectic Al-Si alloys, as indicated by the numerous lighter regions consisting of α-Al in Figure 3a5–7,b6,7,c7,d7. The microstructures observed in this study differed remarkably from those observed in previous reports [20,21], as the microstructures above the Si-rich regions were either eutectic Al-Si or low-Si-content hypereutectic Al-Si alloys. This indicated that our findings could be used to accurately reveal the macrosegregation mechanism of the primary Si phases in hypereutectic Al-Si alloys.

## 4. Discussion

### 4.1. Macrosegregation Mechanism of Primary Si Phase

A previous study [22], which focused on Si segregation, showed that the macrosegregation behavior of the primary Si phases in hypereutectic Al-Si alloys involves a complex coupling of solute distribution, fluid flow, heat transfer, and phase transformation. However, the relative influences of these factors were unclear, thereby limiting the understanding of the macrosegregation mechanism. The experimental results presented herein provide data that can be used to develop a reasonable explanation for Si segregation. Toward this goal, we conducted basic fluid flow and temperature distribution simulations using COMSOL software (5.3, COMSOL Inc., Stockholm, Sweden).

**Figure 4:**
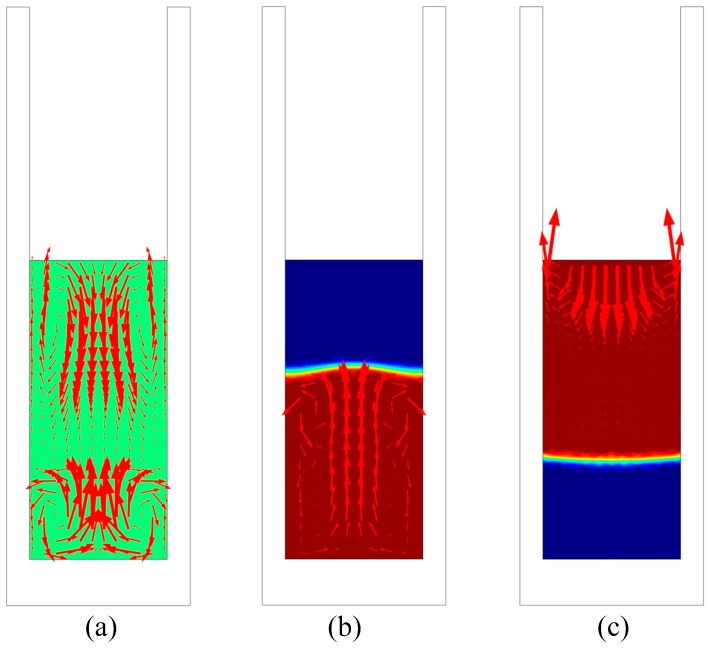
Fluid flow simulation results: (**a**) At the initial sample position, (**b**) after pulling the sample up to a certain position, and (**c**) after pulling the sample down to a certain position during EMDS.

Figure 4 shows the simulation results of the fluid flow during EMDS. Vortex flow (i.e., meridian secondary recirculation flow) [18] (Figure 4a) was induced by the electromagnetic force generated under the alternating electromagnetic field [23]. As the sample was pulled either up or down from the center of the induction-heating zone, the melt flowed intensely because a large volume of molten metal remained in the induction-heating zone, as shown in Figure 4b,c. The intense fluid flow significantly improved the transport capacity in the Al-Si melt. According to the literature [24,25], Si and Si-Si atom clusters were present in the Al-Si melt above the liquids. Therefore, the forced flow promoted the transportation of the Si-rich melt from the remaining liquid melt to the solid-liquid interface. Simultaneously, melt stirring significantly influenced heat transfer and temperature distribution in the melt.

**Figure 5:**
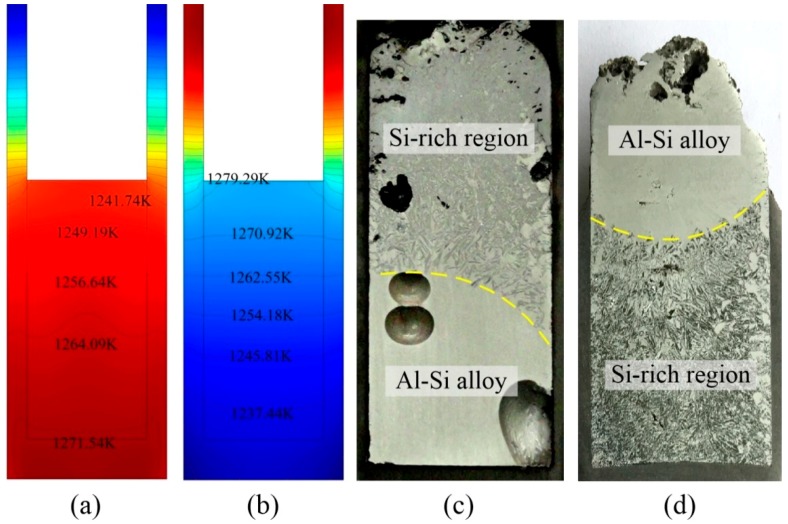
(**a**) Temperature distribution of the sample when pulling up, (**b**) temperature distribution of the sample when pulling down, (**c**) cross-section of the solidified sample when pulling up, and (**d**) cross-section of the solidified sample when pulling down.

Figure 5a,b show the simulation results for temperature distribution during EMDS. It is clear that the temperature decreased from the bottom to top when the sample was pulled up, while it increased from the bottom to top as the sample was pulled down. This implies that a temperature gradient exists during EMDS. Figure 5c,d show cross-sections of the solidified sample while pulling up and down, respectively, showing an opposite direction of Si enrichment. This indicates that the temperature distribution is important for determining the location of the Si-rich layer because the primary Si phase must be preferentially precipitated in the lower temperature region. The rounded pores, as shown in Figure 5c, may be the shrinkage cavities formed during the solidification process.

**Figure 6:**
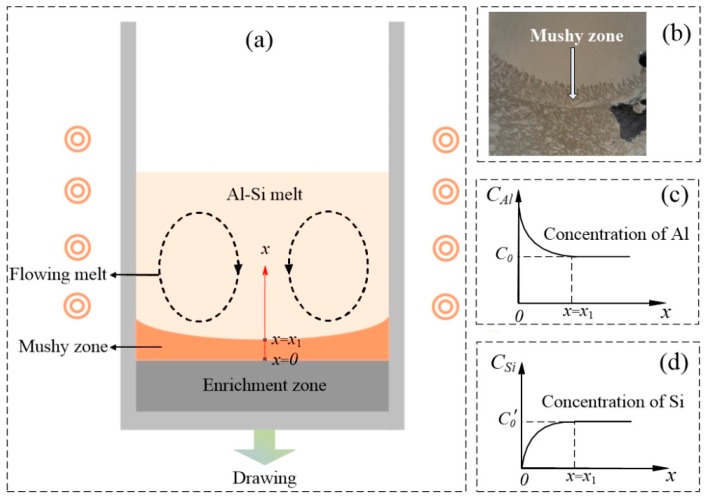
(**a**) Schematic of a plausible Si macrosegregation mechanism, (**b**) cross-section of the directionally solidified sample, (**c**) concentration distributions of Al in the mushy zone, and (**d**) concentration distributions of Si in the mushy zone.

Figure 6a shows a schematic of Si segregation in the hypereutectic Al-Si alloy during EMDS. The sample can be roughly divided into three zones during directional solidification, i.e., the Al-Si melt, mushy zone, and enrichment zone. Upon application of the high-frequency induction heating, the Al-Si melt intensely flowed and carried the Si-rich melt to the solid-liquid interface (i.e., mushy zone), where the Si atoms were rigorously exchanged between the mushy zone and Al-Si melt [22]. Figure 6b shows the experimental results obtained from our previous study [26], confirming that a mushy zone formed at the interface between the Si-rich region and Al-Si melt. Considering the viscosity in the mushy zone is significantly higher than that in the Al-Si melt [26], mass transfer in the mushy zone is likely diffusion controlled. Si atoms and Si-Si atom clusters were transported by the intensely flowing melt to the solid-liquid interface, where they diffused through the mushy zone to Si crystal growth areas. Simultaneously, Al atoms in the mushy zone diffused into the Al-Si melt. According to the boundary layer theory, the concentration distribution of a solute in the mushy zone can be described using an exponential function [27]. Therefore, the concentration distributions of Al and Si atoms in the mushy zone can be described by the functions shown in Figure 6c,d, respectively. It should be noted that the concentration of Al in the remaining Al-Si melt must increase as directional solidification proceeds, while that of Si decreases gradually.

### 4.2. Microstructural Evolution of the Primary Si Phase in Hypereutectic Al-Si Alloys

Considering changes in the Si phase morphology shown in Figure 3, constitutional supercooling theory [28] can be used to explain the observed Si microstructural evolution. Constitutional supercooling is a common phenomenon during solidification and can result in unstable crystal growth at the interface [29]. The underlying mechanism of constitutional supercooling arises from the difference between the actual temperature (depending on the solidification conditions) at a certain point and the liquidus temperature (depending on the solute composition) at the same point. The greater the difference between these two temperatures, the more serious the constitutional supercooling becomes. The constitutional supercooling criterion can be expressed by Equation (1) [28]:

(1) $$\frac{G}{R}\geq \frac{m_{L}C_{0}\left(1-k\right)}{Dk}$$

where *G* is the temperature gradient ahead of the solid-liquid interface, *R* is the Si primary phase crystal growth rate, *m_L_* is the slope of the liquidus line in the Al-Si binary diagram, *C*_0_ is the initial concentration of the Al solute in the remaining Al-Si melt, *D* is the diffusion coefficient of the Al solute in the remaining Al-Si melt, and *k* is the ratio of the Al solute segregated between the directionally solidified Si and Al-Si melt.

If the inequality in Equation (1) is satisfied, constitutional supercooling will not occur. Under these conditions, a planar primary Si phase grows [30], indicating that planar (or bulk) Si can be achieved in hypereutectic Al-Si alloys by properly controlling solidification conditions. If the inequality in Equation (1) is not satisfied (i.e., *G*/*R* < *m_L_C*_0_(1 − *k*)/*Dk*), constitutional supercooling occurs. With increasing degree of constitutional supercooling, the interfacial morphology of the primary Si phase follows a transition through planar → cellular → columnar → and dendritic stages [31]. This is consistent with the experimental results shown in Figure 3.

Based on the above analysis, a clear understanding of the solidification parameters should be necessary to accurately control the morphology of the primary phase Si. From the inequality in Equation (1), the diffusion coefficient of the Al solute (*D*) and segregation ratio (*k*) are nearly unchanged within certain alloy composition and temperature ranges. For instance, *D*_Al_ ranged from 1.65 to 1.70 × 10^−8^ m^2^/s when the concentration of Si in the molten Al-Si alloys ranged from 20 to 40 at.% [32], and k ranged from 5.7 to 7.7 × 10^−3^ when temperature ranged from 1173 to 1373 K [33]. Thus, *D*_Al_ and *k* were considered to be constants for simplification. Additionally, the slope of the liquidus line (*m_L_*) can be considered constant because the liquidus line of Si in Al-Si binary diagram is a gently sloped curve [34]. Therefore, variations in *G*, *R*, and *C*_0_ in Equation (1) are the main factors required for evaluating the degree of constitutional supercooling.

**Figure 7:**
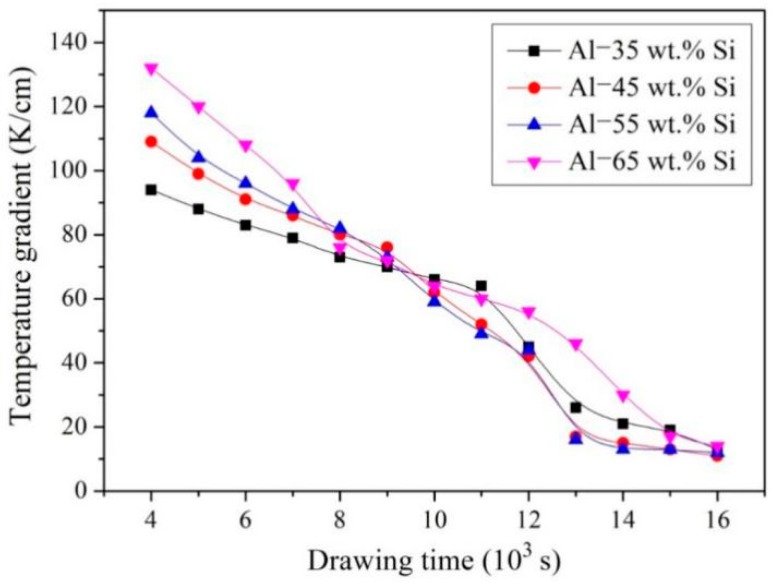
Temperature gradient variation as a function of drawing time.

Figure 7 shows the variation of temperature gradient (*G*) at the front of solid-liquid interface. The measurement of the temperature gradient (*G*) was explained in detail in the experimental section. The temperature gradient (*G*) decreased as drawing the samples out from the induction-heating zone. The decreasing temperature gradient can be attributed to the changing thermal conditions during directional solidification. As the samples were drawn out from the induction-heating zone, the portion of the sample in the cooler zone gradually increased while that in the heating zone gradually decreased. Hence, the cooling rate of the sample changed, which result in the variation of the temperature gradient.

**Figure 8:**
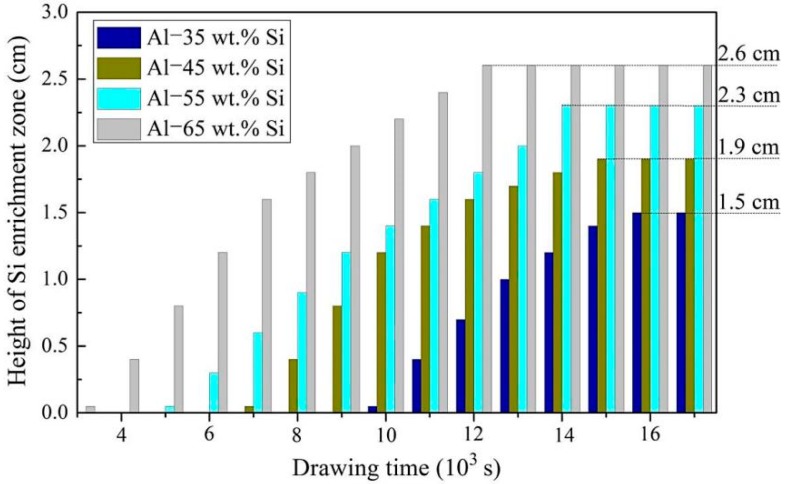
Height change of Si growth interface with the drawing time.

Figure 8 shows the height change of the Si growth interface during EMDS. All the alloys presented a similar growth trends. The Si-rich layer was preferentially formed in Al-65 wt.% Si alloy, and then it appeared in Al-55 wt.% Si, Al-45 wt.% Si, and Al-35 wt.% Si orderly as the drawing time increased. The sequence of Si precipitation should be dependent on the liquidus temperature of the alloys. A higher Si content surely has a higher liquidus temperature [34]. With increasing the drawing time, the height of Si-rich layer gradually increased, and the final heights of Si-rich layer in Al-65 wt.% Si, Al-55 wt.% Si, Al-45 wt.% Si, and Al-35 wt.% Si were 2.6, 2.3, 1.9, and 1.5 cm, respectively.

**Figure 9:**
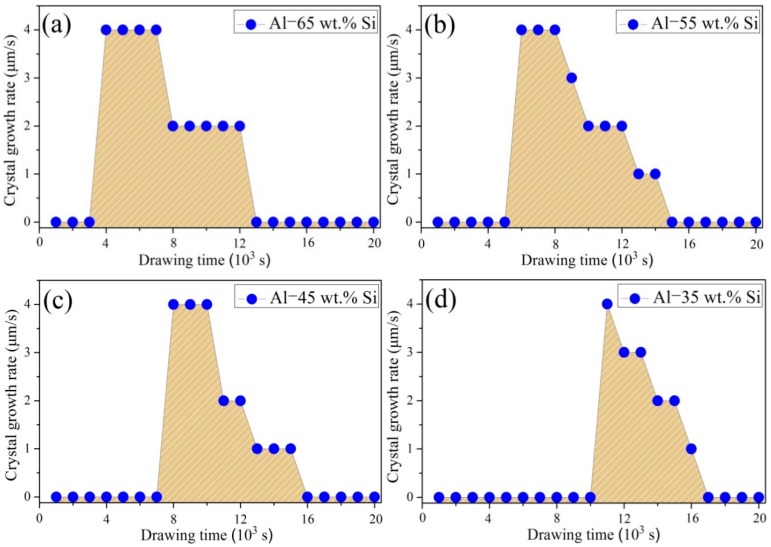
Growth rates of the Si-rich layers: (**a**) Al-65 wt.% Si, (**b**) Al-55 wt.% Si, (**c**) Al-45 wt.% Si, and (**d**) Al-35 wt.% Si.

From the Si growth height and drawing time, the growth rate (*R*) of the Si-rich layer was obtained, as shown in Figure 9, where the growth rate (*R*) decreased during the EMDS. The maximum growth rate (4 μm/s) was slower than that of the drawing velocity (5 μm/s), indicating that the Si crystal growth rate (*R*) in Equation (1) cannot be substituted by the drawing velocity. The change of *R* can be attributed to the variable solidification conditions during the EMDS. Assuming that the Si crystal diffusion is controlled in the mushy zone, the Si crystal growth rate, *R* (m/s), can be calculated using Equation (2), the steady-state diffusion equation [17].

(2) $$R=D_{\mathrm{Si}\;in\;Al–\mathrm{Si}\;\mathrm{melt}\;}\frac{\partial X_{si\;in\;Al–\mathrm{Si}\;\mathrm{melt}}}{\partial x}$$

Here, *D*_Si in Al-Si melt_ (m^2^/s) and *X*_Si in Al-Si melt_ denote the diffusion coefficient of Si and the Si content in the Al-Si melt, respectively. When the alloy is placed in a temperature gradient, ∂*T*/∂*x* (K/m), the Si crystal growth rate can be rewritten as Equation (3).

(3) $$R=D_{\mathrm{Si}\;in\;Al–\mathrm{Si}\;\mathrm{melt}\;}\frac{\partial X_{si\;in\;Al–\mathrm{Si}\;\mathrm{melt}}}{\partial T}\;\frac{\partial T}{\partial x}$$

where *∂X*_Si in Al-Si melt_/∂*T* is the slope of liquidus line in the Al-Si binary diagram and *R* is proportional to the temperature gradient (*G*). Due to the temperature gradient (*G*) decreased during the EMDS, the Si crystal growth rate (*R*) should also decrease.

**Figure 10:**
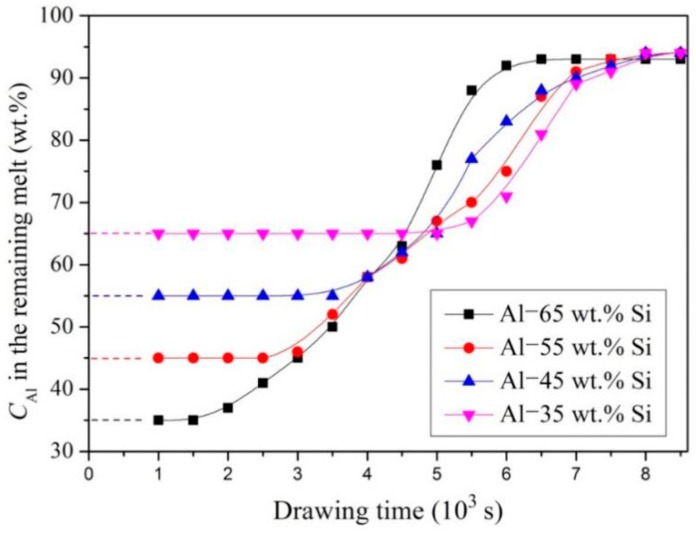
Variation of Al concentration in the remaining melts with the drawing time.

Figure 10 shows the changes in Al concentration (*C_Al_*) in the remaining Al-Si melts during EMDS. Image-Pro Plus 6.0 software (Media Cybernetics, Rockville, MD, USA) was used to determine the volume fraction of the primary Si phase in the bottom region of the Si-rich layer. Thus, Al concentration in the remaining Al-Si melt was calculated by considering mass conservation and the difference between the densities of Al and Si. Figure 10 shows that the Al concentration in the remaining Al-Si melt continuously increased as the primary Si phase precipitated at the bottoms of the samples. The Al concentration above the Si-rich regions (as confirmed using inductively coupled plasma optical emission spectrometry (ICP-OES) by an ICAP 6000 series instrument (Perkin Elmer, Waltham, MA, USA) was ~94%, indicating that this region adopted a typical hypoeutectic composition consistent with the results shown in Figure 3.

The obtained values of *G*, *R*, and *C*_0_ were used to establish a predominance area diagram for determining the Si crystal morphologies according to the inequality in Equation (1).

**Figure 11:**
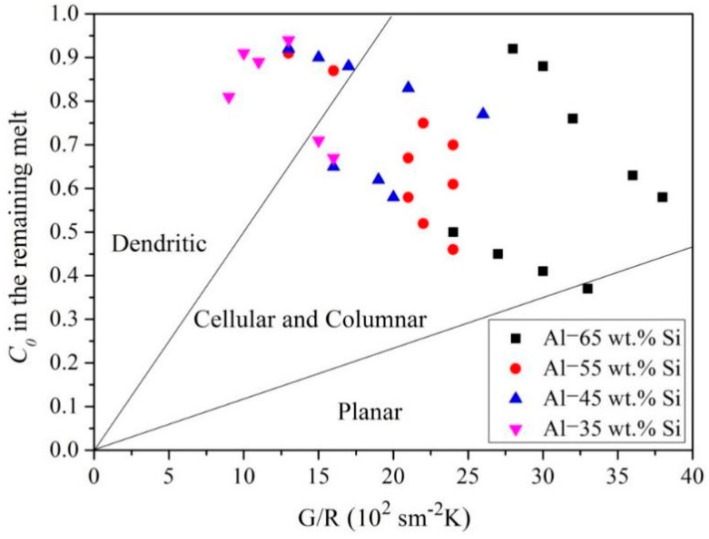
Predominance area diagram for Si morphological evolution.

*G*/*R* and *C*_0_ were regarded as the *x*- and *y*-axes of the diagram, respectively. The Si morphological evolution zone was determined using the obtained values and the inequality in Equation (1), as shown in Figure 11.

Planar Si could be grown by maximizing *G*/*R* or minimizing the Al content in the remaining Al-Si melt. However, it is difficult to satisfy these solidification conditions because the parameters are time dependent. Thus, hypereutectic Al-Si alloys usually exhibit cellular, columnar, or dendritic Si morphologies. Nevertheless, the diagram provides novel insights regarding Si morphological evolution in hypereutectic Al-Si alloys, which can be used to control macrosegregation in alloys for various industrial applications.

## 5. Conclusions

This study investigated the Si segregation behaviors and morphological evolution of primary Si phases in hypereutectic Al-Si alloys. EMDS was used to enhance the macrosegregation of the primary Si phase, and the influences of relevant solidification parameters on the Si morphology were studied in detail. The main findings can be summarized as follows:

(1) The primary Si phase was enriched in hypereutectic Al-Si alloys, forming Si-rich regions during EMDS. The fluid flow induced by electromagnetic stirring played an important role in promoting solute exchange between the mushy zone and bulk melt. In addition, the axial temperature distribution significantly influenced the position of the macrosegregated primary Si phase in the ingots. Thus, the severe macrosegregation of the primary Si phase was mainly caused by fluid flow and temperature distribution.
(2) The morphological evolution of the Si growth interface in the Si-rich region transitioned through planar → cellular → columnar → and dendritic stages during EMDS. The primary reason for the morphological evolution was attributed to the variation in constitutional supercooling ahead of the solid-liquid interface, which was mainly controlled by the temperature gradient (*G*), Si crystal growth rate (*R*), and initial concentration of solute in the melt (*C*_0_).
(3) The experimental results showed that *G* and *R* both decreased while the Al concentration in the remaining A-Si melt (*C*_0_) gradually increased as EMDS proceeded. A method for predicting the variation in Si morphology was established using constitutional supercooling theory and experimental data. By adjusting the cooling conditions (*G*/*R*) and initial solute concentration (*C*_0_), the morphology of the primary Si phases in hypereutectic Al-Si alloys could be accurately controlled.
